# Decoding Metabolic Reprogramming in Plants under Pathogen Attacks, a Comprehensive Review of Emerging Metabolomics Technologies to Maximize Their Applications

**DOI:** 10.3390/metabo13030424

**Published:** 2023-03-13

**Authors:** Ahmed Serag, Mohamed A. Salem, Shilin Gong, Jian-Lin Wu, Mohamed A. Farag

**Affiliations:** 1 Pharmaceutical Analytical Chemistry Department, Faculty of Pharmacy, Al-Azhar University, Cairo 11751, Egypt; 2 Department of Pharmacognosy and Natural Products, Faculty of Pharmacy, Menoufia University, Gamal Abd El Nasr st., Shibin Elkom 32511, Menoufia, Egypt; 3 State Key Laboratory of Quality Research in Chinese Medicine, Macau Institute for Applied Research in Medicine and Health, Macau University of Science and Technology, Macau 999078, China; 4 Pharmacognosy Department, College of Pharmacy, Cairo University, Kasr el Aini St., Cairo 11562, Egypt

**Keywords:** biotic stressors, defense response, mass spectrometry, metabolic reprogramming, metabolomics, plants

## Abstract

In their environment, plants interact with a multitude of living organisms and have to cope with a large variety of aggressions of biotic or abiotic origin. What has been known for several decades is that the extraordinary variety of chemical compounds the plants are capable of synthesizing may be estimated in the range of hundreds of thousands, but only a fraction has been fully characterized to be implicated in defense responses. Despite the vast importance of these metabolites for plants and also for human health, our knowledge about their biosynthetic pathways and functions is still fragmentary. Recent progress has been made particularly for the phenylpropanoids and oxylipids metabolism, which is more emphasized in this review. With an increasing interest in monitoring plant metabolic reprogramming, the development of advanced analysis methods should now follow. This review capitalizes on the advanced technologies used in metabolome mapping in planta, including different metabolomics approaches, imaging, flux analysis, and interpretation using bioinformatics tools. Advantages and limitations with regards to the application of each technique towards monitoring which metabolite class or type are highlighted, with special emphasis on the necessary future developments to better mirror such intricate metabolic interactions in *planta*.

## 1. Introduction

As important part of our ecosystem, plants represent one of the two major living kingdoms. Plants satisfy basic human needs such as food, clothing, shelter, and medicine. Plants produce thousands of specialized metabolites to attract pollinators and to protect from a plethora of environmental stresses [1]. Plant metabolites are considered an indispensable source of staple food as well as a natural source for the pharmaceutical industry [2,3]. Plant metabolites have been shown to manage several human diseases from pain to malaria and cancer [4]. Plants are continuously subjected to diverse stresses such as biotic and abiotic stresses in their life [5,6]. Thus, the proper integration of these inputs is indispensable for optimal growth and fitness [7]. The abiotic stresses includes salinity, drought, radiation, floods, heavy metals and temperature, while biotic stresses include attacks by pathogens such as bacteria, fungi, oomycetes, nematodes and herbivores [8]. As sessile organisms, plants have evolved complex molecular networks to adapt to the diverse stimuli [9]. Although such networks would be beneficial for plant survival by enhancing, for example, resistance against stimuli, they come with reduced growth or yield, a phenomenon known as the ‘growth–immunity tradeoff’ [10]. The complex network of plant hormones regulates these tradeoffs [11].

Pathogen-induced plant diseases, commonly known as phyto-pathogenesis, seriously affect food security and threaten human health [12]. Constant attacks by pathogens, particularly for crops, lead to severe pre- and postharvest losses in yield and cause economic problems. For instance, the fungus *Colletotrichum graminicola* Ces. Wils., the causal agent of maize anthracnose disease, is responsible for annual losses in billions of dollars in the United States [13]. In addition, the devastating disease Fusarium head blight (FHB), caused by the necrotrophic pathogens, affects the quality and yield of several monocotyledonous plants such as wheat, maize, and barley, constituting an economic crisis in many countries [14]. Therefore, the proper understating of molecular mechanisms of plant defense under pathogen attacks is crucial for developing sustainable plant protection programs [15].

Plant pathogens can be divided into two main classes depending on their lifestyle [16]. Some pathogens are biotrophs, where they feed on the infected plant without killing it [17]. Examples include *Magnaporthe grisea*, the causal agent of rice blast disease, and *Pseudomonas syringae*, which causes severe losses in tomato and beans. Necrotrophic pathogens such as *Septoria tritici* and *Erwinia carotovora* secrete effector proteins, toxins, or degrading enzymes, leading to the death of the host plant [18]. Other pathogens are hemibiotrophic, and they initially infect their host as biotrophic before turning necrotrophic, resulting in plant death [19]. *Mycosphaerella graminicola*, *Bipolaris sorokiniana*, and *Zymoseptoria tritici* are hemibiotrophic pathogens that infect wheat [20].

Plants have evolved an intricate defense system for protection against pathogen attacks; however, some pathogens have the ability to parasitize particular plant species [21]. The primary chemical defense mechanisms of plants against most biotic stresses consist of metabolic adaptations [22]. Hence, plant metabolites are involved in resistance and response to pathogen attacks. Thus, the simultaneous evaluation of the metabolome dynamics is essential to deciphering the role of metabolites upon pathogen attacks. The development of genomics, transcriptomics, and proteomics approaches contributed to our understanding of plant–pathogen interactions. In the last decade, the applications of metabolomics greatly improved our understanding of plant responses to pathogens in plant pathology research [23]. Metabolomics applications provide also a snapshot of the plant metabolites in response to pathogens attacks [24,25]. Since metabolites are the end products of biological processes coordinated by genes, transcripts, or proteins, thus, integration of metabolomics with other ‘omics’ approaches is essential for untangling the mechanisms of pathogens attacks. The utilization of model plants such as *Arabidopsis thaliana*, *Solanum lycopersicum*, and *Zea mays* greatly improved our understanding of many plant diseases [26].

In addition, advances in analytical techniques allowed the comprehensive metabolic profiling of plants during pathogen attacks. The marker metabolites for pathogen infection can (1) allow the discovery of novel defense compounds, (2) provide information about plants’ defensive state, and (3) serve as the base for agronomic applications such as crop protection [22].

In this review, the defensive role of specialized metabolites in response to pathogen attacks is elucidated. We do not aim to completely cover every single aspect of pathogens and genes involved in defense mechanisms, as these items have been recently covered [27,28,29,30,31,32,33]. Instead, we focus on the recent advances in the analytical methods for metabolite profiling and data analysis, presenting a comprehensive critical overview on the current tools available for the application of metabolomics, highlighting the potential of each technique in the context of plant–pathogen interactions for the first time in the literature.

## 2. Plant Innate Immunity and Plant–Pathogen Interaction

**Figure 1:**
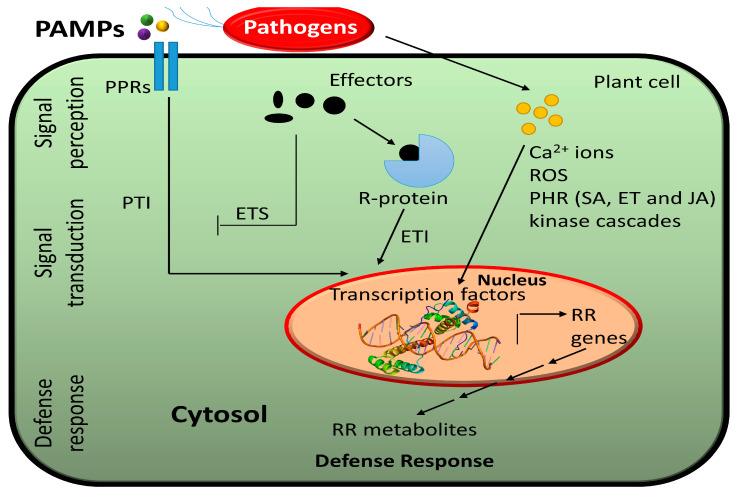
A schematic overview of molecular reprogramming in response to plant–pathogen interaction. Pathogen-associated molecular patterns (PAMPs) are detected by pattern-recognition receptors (PRRs) located in the plasma membrane, inducing pattern-triggered immunity (PTI). Some pathogens produce effectors. Pathogens use their effectors to induce effector-triggered susceptibility (ETS). To stop pathogenesis, plants have evolved resistance (R) genes triggering effector-triggered immunity (ETI) to produce resistance-related (RR) metabolites. Several secondary messengers such as calcium ions (Ca^2+^), reactive oxygen species (ROS), phtyohormone (PHR)-mediated defense pathways as well as several kinase cascades are activated to induce a hypersensitive response or reduce susceptibility.

Plant pathogens infect their hosts though diverse ways. For instance, pathogenic bacteria get an access to the plant cell by entering though stomata or wounds, while aphids and nematodes use their stylets to directly reach the plant cell [34]. Fungi can infect a plant though direct penetration of epidermal cells. To ensure their microbial fitness, pathogens deliver effectors such as virulence factors into the plant cell. Unlike mammals, plants lack mobile immune cells and an adaptive immune system. Instead, plants have an innate immune system that is based on the presence of specific receptors that detect pathogens. During pathogen infection, plants trigger pattern-triggered immunity (PTI) and effector-triggered immunity (ETI) [35] (Figure 1). Pattern recognition receptors (PRRs) within the cell membrane detect pathogen-associated molecular patterns (PAMPs) and during infection, wall-associated kinases (WAKs) detect damage-associated molecular patterns (DAMPs) resulting from cellular damage [31,36]. Receptors with nucleotide-binding domains and leucine-rich repeats (NLRs) detect pathogen effectors [31].

Plants have evolved resistance (R) proteins to recognize pathogen effector and defend through effector-triggered immunity (ETI) [11]. PRRs, WAKs, and NLRs initiate many signaling cascades such as activation of calcium-dependent protein kinases, mitogen-activated protein kinases (MAPKs), transcription factors (TFs), G-proteins, ubiquitin, and hormones [7,34,36]. This leads to various responses such as hypersensitive response (HR), cell wall modification, closure of stomata, production of reactive oxygen species (ROS), production of specific proteins (e.g., defensins, chitinases, protease inhibitors) or production of specific metabolites (e.g., phytoalexins) to protect against further infection [31]. 

### 2.1. Defense Metabolites Aid Plants to Cope with a Plethora of Stressful Pathogens

**Figure 2:**
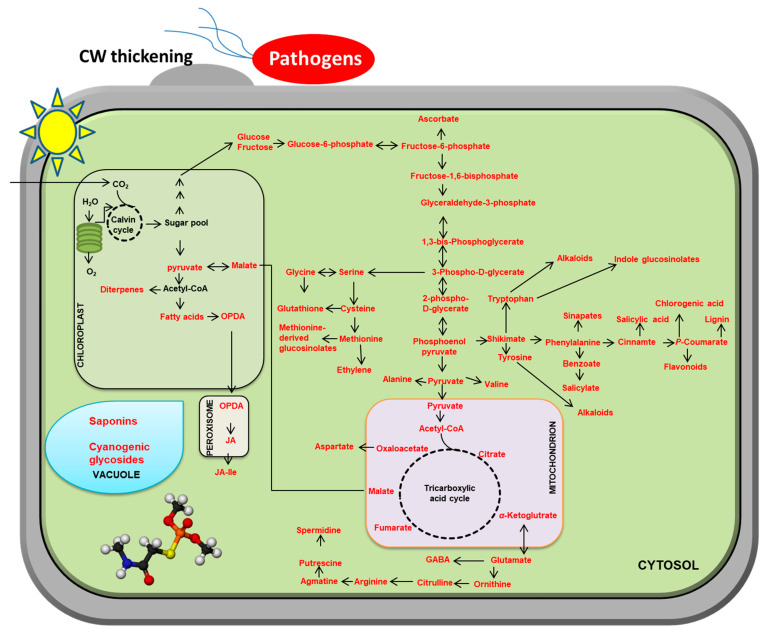
Plant metabolite reprogramming in response to pathogen attacks. The metabolites with red characters represent induction upon pathogens attacks. For simplicity, only some of the main pathways are shown. Solid arrows represent a single reaction or multiple reactions.

Plants produce primary and secondary metabolites [37]. Although they are not essential for growth and development, secondary metabolites are indispensable for the survival of plants, as they help plants to cope with their ecosystems [38]. As sessile organisms, plants cannot escape a plethora of pathogen stresses. Therefore, plants have evolved species-specific diverse secondary metabolites, whose precursors are derived from the primary metabolism [39]. This results in a shift from biosynthesizing essential metabolites for growth to secondary metabolites for defense (Figure 2). 

**Figure 3:**
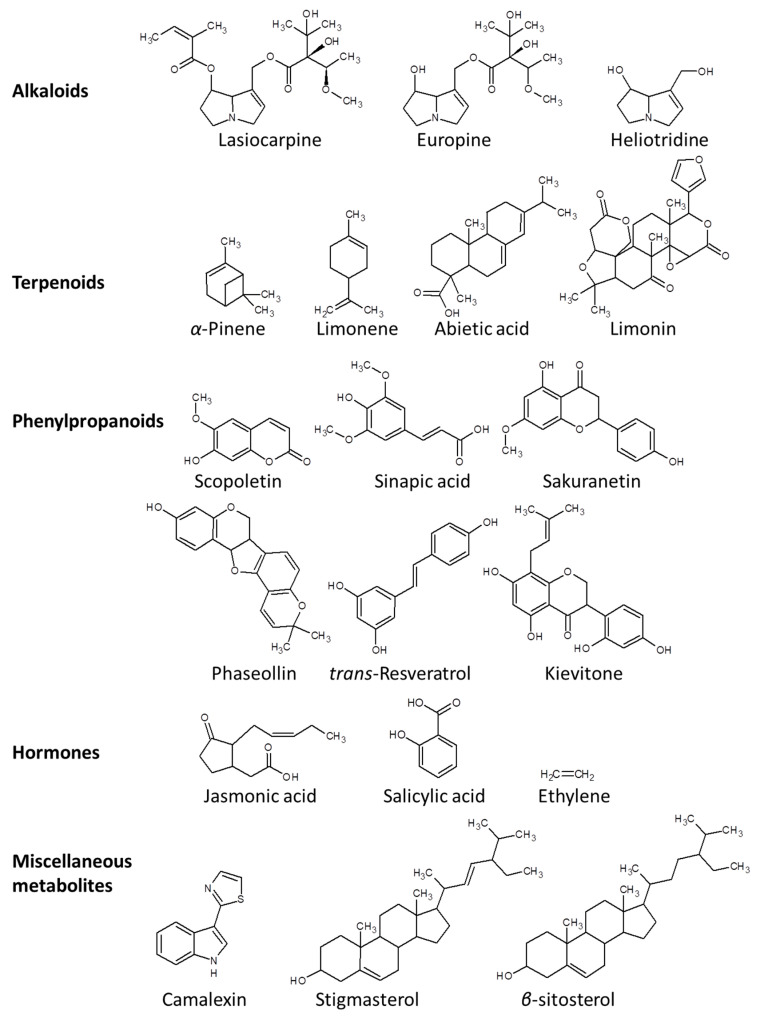
Chemical structures of exemplary metabolites that aid plants to cope with a plethora of pathogens.

The chemical defense arsenal of plants against pathogens can be represented by three major groups of secondary metabolites, namely, alkaloids, isoprenoids, and phenylpropanoids (Figure 3) [9,39]. Alkaloids are nitrogenous compounds that are mainly synthesized via the citrate cycle or shikimate pathway [40]. In particular, pyrrolizidine alkaloids (PAs) such as lasiocarpine, usaramine, europine, monocrotaline, heliotridineand azido-retronecine play indispensable roles primarily in defense against herbivoral attack and microbial infection [41,42].

Isoprenoids are mainly synthesized via methyl erythritol phosphate or the acetate-mevalonate pathway [43]. Monoterpenes (C10) exert potential toxicity against many insects. For example, the monoterpenes esters, pyrethroids, produced by the leaves and flowers of *Chrysanthemum* species, show strong insecticidal activity against wasps, moths, and beetles [44]. Volatile monoterpenes such as *α*-pinene, *β*-pinene, limonene, and myrecene accumulate in resin ducts of several conifers (Gymnosperms) to combat against serious pathogens. A number of sesquiterpenes (C15) such as the lactone derivatives costunolides were reported as antiherbivore agents produced by several plants of the family Asteraceae. Diterpene resin acids (C20) such as abietic acid and its derivatives have been shown to confer resistance against herbivory and pathogen attacks [45]. Among trierpenes (C30), limonoids possess strong insecticidal, nematicidal, and antifungal activities [46]. Emerging evidence demonstrates that plant volatile emissions (e.g., volatile organic compounds) constitute a major fraction of plant defense metabolites. Volatile methyl esters of anthranilic acid have been associated with defense against insect herbivores [47]. In addition to their role as antimicrobial compounds, plants can also produce herbivore-induced volatiles for the recruiting of natural predators of herbivorous insects [48]. For instance, maize leaves produce terpenoid and indole compounds in response to infection by caterpillars for attracting wasps that feed on caterpillars [31]. Plants can also protect themselves from insect attacks via production of sticky metabolites such as latex or resins that trap insects [49].

Phenylpropanoids, phenylalanine derivatives with a basic C6-C3 skeleton, are derived from the plastidic shikimate pathway [50]. Phenylalanine, which is synthesized from the shikimate pathway, can be converted through the phenylpropanoid biosynthesis pathway into aromatic compounds, including flavonoids, hydroxycinnamates, coumarins, benzenoids, and lignin [51]. These compounds are involved in plants’ defense system against pathogenic infection [52]. For instance, chlorogenic and sinapic acids have been proven to have an efficient defense against a wide range of insect herbivores [53]. Lignin, which is biosynthesized via the phenylpropanoid biosynthesis pathway, is composed of phenolic heteropolymers resulting from the oxidative coupling of *p*-coumaryl, coniferyl, and sinapyl alcohols. The cross-coupling reaction of these monolignols forms phydroxyphenyl, guaiacyl, and syringyl lignins [54]. Interestingly, lignans, which serve as a storage pool of monolignols, also play functional roles against pathogen via inhibiting pathogenic degrading enzymes such as cellulases, glucosidases, and polygalacturonases [55]. Lignins and lignans prevent pathogen spreading by enforcing the secondary cell walls [56].

Several miscellaneous metabolites have also been reported to combat against pathogens. Phytosterols such as stigmasterol and *β*-sitosterol play key roles in defense against many pathogens [57]. Callose, which is a *β*-1,3-glucan polymer, is deposited around the hyphae of several biotrophs leading to resistance against the infected pathogen. This resistance has been reported against the powdery mildew fungus *Blumeria graminis* in barley and wheat [58]. Some plant hormones are among the key plant defense metabolites involved in plant–pathogen interaction to ensure the resilience and tunability of plant immunity [7]. Long-distance signaling modulated by hormones enables plants to acquire long-term adaptation to diverse biotic stresses [59]. In particular, jasmonates (JAs), salicylates (SAs), and ethylene (ET) have been considered the primary defense hormones against pathogens [60]. Biotrophic pathogens and sap feeders generally induce the SA pathway, while insect chewing induces the JA pathway [61]. Necrotrophic pathogens trigger both ET and JA pathways [62]. Allantoin, a purine metabolite that activates JA signaling, has been shown to be induced upon pathogens attack, particularly by the fungus *Trichoderma harzianum* [61]. Most phospholipids, such as phosphatidic acid, phosphoinositides, lysophospholipids, sphingolipids as well as oxylipins, can be rapidly activated upon pathogens attack [63].

### 2.2. Phytoanticipins, the Constitutive Chemical Barriers

Beside the physical barriers such as cell walls, plants have effective pre-formed chemical defense secondary metabolites termed phytoanticipins, representing the first layer of defense upon pathogen attacks [64]. Among the diverse phytoanticipins, cyanogenic glycoside, saponins, and glucosinolates represent the major classes of defense metabolites [31].

Structurally, cyanogenic glycosides are characterized by presence of the α-hydroxynitrile (cyanohydrin) group that is stabilized by glucosylation [65]. Cyanogenic glycosides are not toxic on their own. When the plant cell structures are disrupted by herbivores, cyanogenic glycoside is subjected to hydrolysis by the corresponding β-glucosidase, releasing the toxic hydrogen cyanide (HCN) [65]. Saponins are glycosylated compounds that are widely distributed in some plant families. According to aglycone nature, steroidal and triterpenoidal saponins exist. The genus Avena, particularly oats, contains the steroidal avenacosides accumulated in leaves and triterpenoid avenacins accumulated in roots [66]. The resistance of oats to fungal infections such as those induced by *Gaeumannomyces graminis* is attributed to avenacins contents [64]. Another saponin in tomato, α-tomatine, is correlated with the resistance to *Fusarium oxysporum*, *Verticillium albo-atrum*, and *Cladosporium fulvum* [67].

Glucosinolates (S-glucopyranosyl thiohydroximates) are sulfur-containing glycosides that are commonly distributed among plants of the family Brassicaceae [68]. Structurally, glucosinolates have *β*-thioglucose moiety, sulfonated oxime moiety, and aglycone side chain. According to their side chain, glucosinolates may be derived from aliphatic (methionine), indolyl (tryptophan), or aralkyl (phenylalanine) α-amino acids [68]. Like cyanogenic glycosides, glucosinolates are spatially compartmentalized from the enzymes that degrades them [69]. The resistance of Brassica to the fungus *Leptosphaeria maculans* and the oomycete *Peronospora parasitica* has been attributed to glucosinolates content [70]. Additionally, cabbage and cauliflower leaves resist the attacks by *Mycosphaerella brassicae* through their glucosinolates contents [71].

### 2.3. Phytoalexins, the Inducible Antimicrobial Metabolites

In addition to the pre-formed defense metabolites, plants also possess chemical defenses, known as phytoalexins, which are only induced upon pathogens attacks or synthesized ex novo [72]. Pathogens trigger the activation to enzymes involved in the phytoalexin biosynthetic pathways, leading to an unspecific inhibitory effect against a wide range of pathogens [73,74]. Activation of plant defense response induces transient metabolic reprogramming to the direction of de novo synthesis of phytoalexins from primary metabolites amino acids, coenzyme A derivatives, and mevalonic acid [39].

A classical phytoalexin produced by the model plant *Arabidopsis thaliana* in response to many pathogens is camalexin (3-thiazol-2’-yl-indole)[75]. The production of this compound is regulated by mitogen-activated protein kinase (MAPK) and WRKY transcription factors (TFs) [76,77]. Among pathogens that induce camalexin production are fungi such as *Alternaria brassicicola* and *Botrytis cinerea* as well as bacteria such as *Pseudomonas syringae*. Viruses and oomycetes were also reported to induce camalexin production [78].

Isoflavonoids from the Leguminoseae family have been shown to have a defensive role as antibacterial phytoalexins against a wide range of pathogenic microorganisms [79]. *Phaseolus vulgaris* has been shown to produce phytoalexins such as phaseollin and kievitone in response to pathogen attack by the hemibiotrophic fungus *Colletotrichum lindemuthianum*, the causal of the bean anthracnose [80]. Additionally, the isoflavonoids medicarpin from *Medicago sativa* and pisatin from *Pisum sativum* represent similar examples from the Leguminoseae family.

Other plant families such as the Vitaceae produce phytoalexins with a stilbene-derived skeleton in response to fungal attacks. For instance, the glucosides derivatives of *trans*-Resveratrol (3,5,4′-trihydroxystilbene) known as piceids as well as the dimethylated derivatives (pterostilbenes) and oligomers derivatives (viniferins) were also reported [78]. Scopoletin (6-methoxy-7-hydroxycoumarin) is the major phytoalexin that is produced by tobacco leaves in response to pathogen attacks, particularly by *Alternaria alternate* [81]. Additionally, Sakuranetin (derivative of the flavonoid naringenin) and the diterpenoid momilactone have been shown to protect rice from pathogenic attacks by the fungus *Pyricularia oryzae*, the causal agent of rice blast, which can causes severe yield losses [64].

### 2.4. Pathogenesis-Related Omics Data Provide New Findings to Study Plant Defense Responses

Since pathogens impair plant growth and reproduction, fundamental studies on the plant immune system play an indispensable role in improving our knowledge of plant resistance to pathogens. Pathogen stresses trigger a wide range of plant responses such as alteration in gene expression, translation, and cellular metabolism, and therefore, analysis of such alterations is essential to understanding plant–pathogen interactions. High-throughput omics technologies have been widely used to study the metabolic reprogramming during the process of plant defense response to pathogens [82]. Several studies have been devoted to studying transcriptional reprogramming in response to pathogen attacks [83]. Transcriptome data provide great opportunities to investigate the molecular mechanisms of plant immunity in response to pathogen infections [84,85]. Gene differential co-expression analysis remains the most commonly used method to generate transcriptome data related to plant defense responses. The effective way to analyze these data is to trace the differentially expressed genes (DEGs) as candidates for plant immunity responses. These candidates include cell type-specific and organ-specific genes [36]. Therefore, spatial and temporal resolutions facilitated our understanding of plant–pathogen interactions. The main repositories of high-throughput gene expression data include GEO, ArrayExpress, Expression Atlas, and GEM2Net. Particularly, PathoPlant, ExPath, and PlaD are specific to transcriptome databases related to plant immune response. The analysis of plant proteomes depends on the existence of complete genome sequence databases for the species of interest [86]. Using proteomics approaches, defense-related proteins expressed during phytopathogenic interactions have been identified and extensively reviewed [87,88].

### 2.5. Metabolomics as Better Tools for Decoding Pathogen–Plant Interactions

Metabolomics, the comprehensive, nonbiased, high throughput analysis of the whole set of metabolites within a biological system, originated from the metabolite profiling approach introduced by the pioneering work of the Horning group from the Baylor College of Pharmacy [89]. With the advent and rapid development of mass spectrometry-based analytical techniques, the concept of the ‘metabolome’ was introduced by Oliver et al. in 1998 [90]. Thereafter, the concept of metabonomics, which represents tracing metabolic changes in response to pathological stimuli or genetic modification, was proposed in 1999 [91]. Later, Fiehn proposed the first more detailed definition of metabolomics and its applications in plant biology research [92]. Acting as a bridge between genotypes and phenotypes, a lot of research has been conducted in the area of applying metabolomics in plant biology research for metabolic engineering, crop improvement, food assessment, as well as in clinical research for disease diagnosis, drug screening, and treatment [93,94]. 

Metabolomics has emerged as a functional genomics tool for understanding complex interaction within a biological system. Since metabolites are the end products of the cellular metabolism, and their levels can be seen as an ultimate response of the biological system to external stimuli [92]. Thus, metabolomic studies directly reflect the phenotypic changes of a particular system compared with genomics or proteomics that do not always have a direct correlation to biochemical phenotypes [95]. The changes in the genome, transcript, or proteome of a particular cell will surely produce changes in the metabolome [96]. Since it reflects both transcriptional and post-transcriptional regulation, metabolomics can be considered the ultimate level of post-genomic analyses. Unlike transcripts and proteins, metabolites are diverse in their chemistry, and hence, their determination relies on sophisticated instrumentation such as mass spectrometry (MS) and nuclear magnetic resonance spectroscopy (NMR) [93,97]. 

The common strategies in metabolomics studies include untargeted and targeted approaches [93]. The goal of untargeted metabolomics is to detect as many metabolites as possible from analyzed samples. This approach is hypothesis-generating and well suited for unknown discovery. On the contrary, targeted metabolomics is a biased and hypothesis-driven approach which targets the quantification of a previously determined set of specific metabolites or a certain class of metabolites.

**Figure 4:**
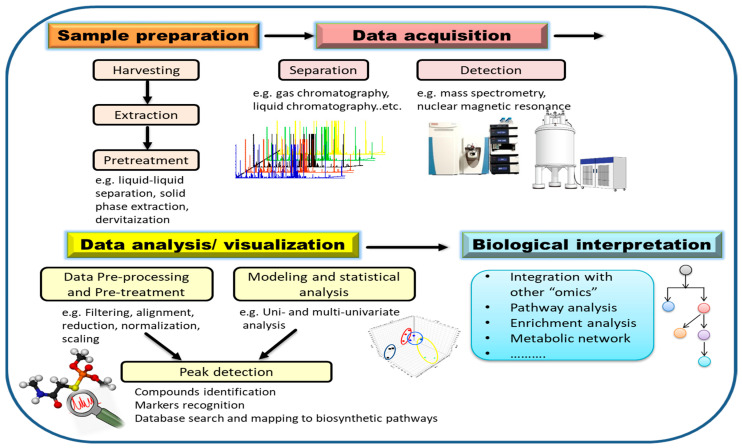
Key steps of a metabolomics study for assessing pathogen–plant interactions.

Steps for metabolomics analysis typically include experimental design, sample preparation, data acquisition, data processing, and biological interpretation (Figure 4). In order to obtain meaningful results, experimental design should be carefully considered. To statistically reflect the validity of the experimental data, factors such as time of harvesting, type of tissue, number of groups, as well as the number of replicates per condition have to be carefully determined [82]. Appropriate sample harvesting, rapid quenching, and direct extraction are highly necessary to avoid metabolite degradation. Extraction of metabolites is an important step in sample preparation that will also dictate which analytes are detected later on based on solvent type and should be carefully considered in a metabolomics investigation. The choice of extraction solvent and method greatly influences the resulted metabolite matrix. Since the diversity of metabolites leads to different solubility, it is difficult to extract all metabolites from a sample within a single extraction method [93]. Therefore, it is necessary to choose the extraction method according to the purpose of the experiment and the range of metabolites to be covered. The universal solvent, water, can efficiently extract primary metabolites such as amino acids, sugars and organic acids. For secondary metabolites, single solvents such as methanol or mixtures of immiscible organic solvents can be used [98]. In the next sections, we focus on the advances in analytical techniques and data analysis. 

## 3. Current Analytical Tools for Studying the Metabolomics Reprogramming in Pathogen—Plant Interactions

### 3.1. NMR Based Metabolomics Analysis

**Figure 5:**
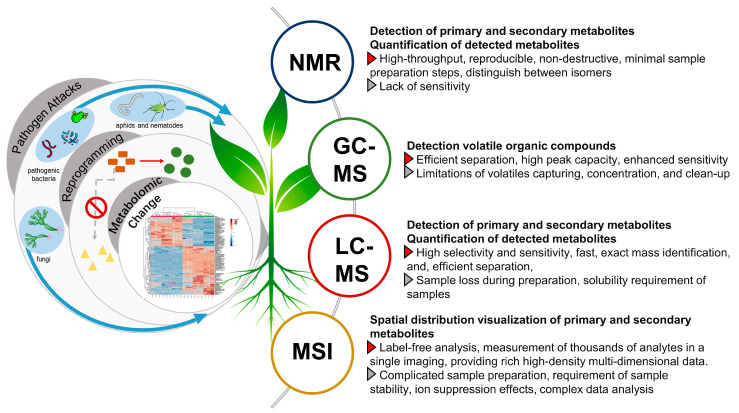
Platforms used for studying the metabolomics reprogramming in pathogen–plant interactions.

NMR is a powerful high-throughput analytical technique that is highly reproducible, non-destructive, and universal with minimal sample preparation steps [93]. Hence, NMR-based methods have been widely used to decode the metabolome that might be implicated in the plant defense against pathogen attacks (Figure 5). Most applications have used the ^1^H over other spin-active nuclei such as ^13^C or ^15^N due to the omnipresence of hydrogen atoms in organic molecules, higher detection level allowing the detection of the abundant primary metabolites, vis., amino acids, organic acids, and sugars. For example, ^1^H-NMR metabolomics analysis identified 11 primary metabolites involved in the chemical defense mechanisms of tomato to bacterial wilt disease caused by *R. solanacearum,* a pathogen that can cause wilting, chlorosis, and death of tomato plants [99]. Of all these metabolites, valine and leucine had a significant difference between resistant and susceptible cultivars. ^1^H-NMR metabolomics analysis also revealed the upregulation of γ-aminobutyric acid and trigonelline in different genotypes of tomato after exposition to *T. absoluta* infection [100]. Such infection can cause up to 80–100% yield losses in tomato crops. 

Considering the complexity of NMR signal overlap especially in the aliphatic region, 2D NMR experiments are considered to aid in identification and resolving signals along the carbon dimension, as explained in the next paragraph [101].

A main advantage of NMR is also its capability to detect plants’ secondary metabolites, being especially more related to stress response, [102] alongside primary metabolites at equal response. Although this approach is not feasible via the one-dimensional ^1^H-NMR due to the crowding and overlap of the signals, various two-dimensional techniques (2D-NMR) have been developed to tackle this problem through spreading the crowded signals in a second frequency domain via magnetization transfer [103]. Such a transfer could be between same type nuclei as in H–H correlated spectroscopy (COSY) and H–H total correlated spectroscopy (TOCSY) or different types of nuclei such as heteronuclear multiple bond correlation (HMBC) and heteronuclear single quantum coherence spectroscopy (HSQC) experiments. For example, 2D-NMR analysis improved the resolution of ^1^H-NMR spectra, allowing the characterization of some phenylpropanoids such as chlorogenic acid, 3-O-caffeoyl quinic acid, and feruloyl quinic acid, posing their role as a resistance factor for thrips in chrysanthemum [104]. The joint analysis of 1D/2D NMR also revealed several metabolites responsible for resistance of *Annona muricata* roots to the nematode *Meloidogyne javanica* including dopamine, xanthine, and aromatic compounds [105]. NMR-based metabolomic profiling of potato leaves also revealed the upregulation of malic acid, methanol, and rutin and downregulation of sucrose in the resistant cultivars to late blight disease more than in the susceptible ones [106]. NMR Metabolomics analysis was also useful for understanding plant–pathogen interactions in citrus canker, a disease caused by *Xanthomonas axonopodis* bacteria. By utilizing transgenic sarcotoxin-expressing sweet orange, this study proved the protective activity of sarcotoxin against infection [107]. These cases demonstrate the potential of NMR-based metabolomics analysis for marker-assisted screening of new cultivars with durable resistance to various infections.

Another major advantage of NMR is that the signal intensities are directly proportional to the corresponding real molar levels of the detected metabolites, which makes NMR an absolute quantitative method without any need for calibration curves of individual analytes as typical and needed in LCMS- and GCMS-based approaches [108]. Quantitative NMR (qNMR) metabolomics analysis allowed the detection of potential metabolic markers in wheat subtypes following *Fusarium graminearum* infection [109]. qNMR also has provided a novel insight for studying the kinetic variations of metabolites of tomato xylem sap infected with the bacterial wilt disease. Glutamine and asparagine were identified as primary resources consumed during the colonization phase and putrescine during the bacterial growth phase of this disease [110]. 

Nevertheless, the lack of sensitivity is one of the major disadvantages of NMR compared to MS (several orders or magnitude lower than that of MS) especially if monitoring phytohormones that are typically present at all levels *in planta* [111]. Although this problem can be partially improved via the use of cryogenic and/or micro probes in addition to the developments in the superconducting magnets, mass spectrometry-based metabolomics are still dominating, particularly in metabolomics studies involved in the revealing of plant–pathogens interactions.

### 3.2. GC-MS Based Metabolomics Analysis of VOCs and Primary Metabolites

Volatile organic compounds (VOCs) have been identified as signaling metabolites involved in both intra- and inter-plant communication, especially in responding to various biotic and abiotic stresses [112]. Of all metabolomics analysis approaches used for scrutinizing the VOCs, gas chromatography hyphenated to mass spectrometry (GC-MS) is the de facto analytical technique employed for such profiling (Figure 5). The principle of GC-MS is driven by three fundamental features, including samples’ volatility, peak capacity, and selectivity. Hence, GC-MS-based metabolomics approaches have been extensively employed in volatile profiling of plant–pathogen interactions posing their role in plant defense mechanisms. GC-MS have been applied to differentially analyze the volatiles emitted in the immune response of the Rio Grande tomato leaves after infection with virulent and avirulent strains of *Pseudomonas syringae* [113]. Seventy-three VOCs have been identified, of which esters of (Z)-3-hexenol and several hydroxylated monoterpenes such as linalool, α-terpineol were upregulated in the infected samples with avirulent strains. Contrarily, infected samples with the virulent strain of *Pseudomonas syringae* produced a different VOCs profile characterized by salicylic acid (SA) derivatives and monoterpenes. GC-MS also has been used to assess the volatiles profile of *C. camphora* callus in comparison to its leaves and in response to methyl jasmonate (MeJA) elicitation with upregulation of different ionone derivatives in the elicited samples, and these compounds were unreported and novel in that genus [114]. Similarly, GC–MS metabolomics analysis of MeJA-elicited *P. minus* leaves identified time-course changes in VOCs that occurred after elicitation with activation in the biosynthetic pathways for aldehydes and terpenes [115]. In addition, GC-MS analysis has been also employed for monitoring and early detection of stored-grain insect infestation via detection of VOCs emitted by insects such as *Tribolium castaneum* (Herbst), *Rhyzopertha dominica* (Fabricius), and *Sitophilus granarius* (Linnaeus) [116]. 

Besides, primary metabolites can be also analyzed by GC-MS post chemical derivatization with silylation reagents such as *N*-methyl-*N*-trimethylsilyltrifluoroacetamid (MSTFA) to increase their thermal stability and volatility. Such an approach has been applied for analyzing primary metabolites involved in the metabolic reprogramming of rice in response to infection by the biotrophic pathogen *Magnaporthe grisea* such as the amino acids proline, tryptophan, histidine, and cysteine along with the glucose, fructose, and sucrose [117]. Moreover, GC-MS has been employed for analyzing the primary metabolites responsible for the resistance of soybean to the cyst nematode *Heterodera schachtii*. Results suggested that the infection was associated with induction of various nematicidal metabolites, including 4-vinylphenol, methionine, piperine, and palmitic acid, which are involved in lignin production and thus, cell wall reinforcement against pathogen penetration [118]. GC-MS-based metabolomics analysis of soybeans (*Glycine max*) in response to infection by the oomycete pathogen *Phytophthora sojae* revealed that several sugars, organic acids, amino acid derivatives as well as secondary metabolites such as daidzein and hypoxanthine participate in defense response [119].

Comprehensive two-dimensional gas chromatography (GC × GC) is another emerging technology used for VOCs profiling [120]. The setup of GC × GC is based on placing two columns in series using a transfer device called a modulator. Those columns are usually packed with stationary phases of substantially different selectivity to the sample’s components, thus warranting better separation, higher peak capacity, and enhanced sensitivity of the system. The main mass analyzer hyphenated with GC × GC setup is the time-of-flight (TOF) mass spectrometer which can acquire 50 or more full mass spectra per 100 ms peaks, an acquisition rate which is more than enough to reconstruct the second-dimension chromatograms. Since all ions are collected, virtually, at the same time points during the full MS acquisitions, the ion ratios remain the same across the GC peaks, resulting in non-skewed chromatograms. Besides, this spectral continuity warrants deconvolution of overlapping GC peaks based on the difference of the fragmentation patterns of the coeluting compounds [121]. Furthermore, TOF MS technology can acquire high mass resolution (5–10 ppm) spectra but with a significant limitation on the acquisition speed. The hyphenation of GC × GC with TOF MS results in high separation power based on the combined use of chromatographic and mass spectral resolutions. Such an instrument is well suited to resolve complex mixtures of analytes such as VOCs since these compounds, most likely, will not have identical retentions times in both GC dimensions (different stationary phases) and identical mass spectra as well. GC × GC-TOFMS has been employed for the metabolic profiling of plant–fungus interaction in *Aquilaria malaccensis* detecting various phytoalexins as a defense system of the host plant in addition to revealing the detoxification mechanisms by the fungus to overcome the plant defense signals [122]. In addition, the interaction between wounded *Nicotiana attenuata* leaves and *Manduca sexta* oral secretions has been also investigated using a GC × GC-TOFMS metabolomics-based approach [123]. Approximately 400 analytes were detected, of which several primary metabolites such as fatty acid and amino acid conjugates were found to play a central role in the plant defense against the pathogen attack. 

However, one of the major challenges in analyzing VOCs using GC-MS and GC x GC-MS is the capturing, concentration, and clean-up of these volatiles. Certainly, solid phase microextraction (SPME) is the dominant sample preparation technique for volatile profiling, since its invention in 1990 [124], attributing to several advantages including simplicity, reusability, reproducibility, and potential incorporation into GC auto-samplers. SPME uses a fiber-based substrate coated by a thin sorbent layer that is exposed to the sample matrix either via direct immersion (DI) or headspace (HS). Analytes pre-concentration is achieved based on their diffusion from the sample matrix into the coating material according to their distribution coefficient. Afterwards, the fiber and its coat is pulled into a protecting needle, and to expose it inside the heated injection port of GC instruments for analytes desorption. Nevertheless, SPME stationary phase type can affect volatiles type adsorption affecting detected volatiles later on using GCMS warranting for the use of more than one coating to ensure comprehensive coverage of the aroma of the targeted plant [125]. Additionally, HS-SPME might now allow for the in situ real time monitoring of VOCs release with biotic interactions as the plant part is typically cut and placed inside the vial for VOCs collection. HS-SPME coupled to GC-MS was used to highlight the interactions between healthy and *Xylella fastidiosa*-infected olive trees based on their VOCs profiles identifying different methyl esters compounds as infection biomarkers and putative diffusible signal factors [126]. In contrast, dynamic headspace (DHS) sampling, also called “purge and trap”, is another sampling technique that can better allow for the in situ monitoring of VOCs release in which the analytes are extracted by purging the sample with a flow of carrier gas into a trap with high retention power either by cryofocusing or on sorbent material [14]. The trapped analytes are then heated or washed with organic solvent later on to release VOCs and released into the chromatographic system with much better detectability than static head space approaches. Such a set up has been coupled with GC-MS for untargeted screening of volatiles emitted by lavender grown under open-field conditions in response to the yellow decline disease [127].

### 3.3. LC-MS Based Metabolomics for Decoding Secondary Metabolites Reprogramming

Liquid chromatography coupled to mass spectrometry (LC–MS) is the most-important method for plant metabolomics, being especially suited to profile secondary metabolites more involved in plant pathogens interaction (Figure 5). Compared to GC-MS, it is more suitable to analyze polar and non-volatile metabolites belonging to secondary metabolites more likely to function as phytoalexins or phytoanticipins, and moreover, the derivatization step is not necessary in LC-MS analysis [128,129]. Moreover, the high selectivity and sensitivity benefit the profiling of phytoalexins and phytoanticipins with low abundance, which may play a chief role in metabolic reprogramming [130,131]. This poses LC-MS as an indispensable analytical tool in monitoring plant metabolic reprogramming in pathogen–plant interactions, and surely other stress responses.

There are a large number of secondary metabolites belonging to various classes such as alkaloids, flavonoids, terpenoids, etc. [52,132], with various molecular weight, polarities, and stabilities, involved in plant metabolic reprogramming. Therefore, an efficient separation of metabolites from complex mixtures can significantly benefit the determination of them. Column technology is a basic choice in LC systems to increase separation capability. The majority of LC–MS applications use C18 reversed-phase (RP) separation, and secondary metabolites determination, especially, typically employ this strategy. C18-based RP separation can separate different types of metabolites (with diverse mass weight and polarity) well in a simple run, convenient for the metabolomic analysis. The influence of the soil biotic legacy to the diversity of secondary metabolites that a plant produced has been revealed by the work of Ristok et al. [133]. Results revealed that quinic acid/quinic acid derivatives, chlorogenic acid derivatives, flavonoid glycosides, verbascosides and iridoid glycosides in *C. jacea* and *P. lanceolata* played an important role in plant–herbivore interactions. Lee et al. analyzed the primary and secondary metabolites of *Schisandra chinensis* by GC/MS and LC/MS, respectively [131]. In this study, 31 secondary metabolites were detected using LC/MS and the differences of these secondary metabolites showed significant relation with their origins. Zhang et al. also described a multi-omics and UHPLC-MS determination of the difference in terpenoids metabolite content during the development of *C. cathayensis* seeds [134]. Besides the C18 stationary phase, examples using other stationary phases such as high strength silica (HSS) T3 in RP mode can also be found. The variation of specialized secondary metabolites triggered by lipopolysaccharides in *Arabidopsis thaliana* was determined using HSS T3 for separation, including phytohormones salicylic acid, jasmonic acid, and the associated methyl esters and sugar conjugates [135]. The defensive state induced the increase of a series of metabolites involved in dynamic reprogramming of metabolic pathways, such as indole and indole derivatives, glucosinolates, camalexin as well as cinnamates and other phenylpropanoids. Other separation modes including hydrophilic interaction chromatography (HILIC) and pentafluorophenyl (PFP) columns are also used for secondary metabolites determination as an alternative stationary phase to RP liquid chromatography. A HILIC system can achieve proper retention for highly polar compounds, such as phenolic acids, flavonoids, and terpenoids, exhibiting very poor retention in RP chromatography and achieving a better separation of them [136]. In addition, PFP is suitable for the separation of broad range phenolic compounds and has a better separation efficiency than C18 columns [137].

However, there is a wide variety of metabolites that are involved in plant defense. Some metabolites are difficult for achieving suitable retention in the LC system or with poor ionization efficiency, preventing the detection of such metabolites. Therefore, other strategies are employed in the LC-MS system for better separation and determination of these metabolites, such as ion pairing, chemical isotope labeling, and derivatization. Ion pairing techniques add ion pairing reagents in the mobile phase to reduce the coeluted ion charged compounds and improve chromatographic performance [138]. In this case, ion-pair chromatography is used for many polar metabolites’ detection in plants, such as alkaloids, polyamines, etc. [139,140,141]. For instance, polyamines played an important role in plant–pathogen interaction [140]. Nevertheless, polyamines are minor compounds, and interferences from complex matrices prevent their accurate determination *in planta*. With the addition of ion-pairing reagent heptafluorobutyric acid (HFBA), the variation of Put, Spd, Spn, and 1,3-diaminopropane in *Arabidopsis thaliana* samples was determined [140]. Moreover, the better separation performance also aids in the identification of novel metabolites of potential defensive action. With the separation using ion-pair forming HFBA, three new acylated betacyanins were newly reported from the Amaranthaceae family, first detected in *I. lindenii* leaves [141].

**Figure 6:**
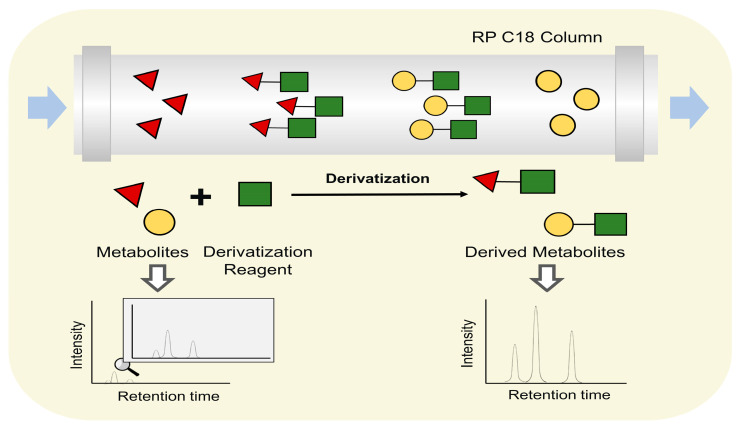
The principle of derivatization approach.

The derivatization technique is another technique to improve separation performance or ionization effect. Derivatization reagents are used to change metabolites polarity or introduce readily ionizable groups to reduce difficulties in their ionization (Figure 6). For instance, brassinosteroids (BRs) is a class of steroid plant hormones which undergo profound changes during plant–pathogen interactions and may take part in metabolite reprogramming [142]. However, the determination of BRs is difficult due to the lack of electrosensitive or easy ionizable groups. Consequently, Wang et al. established an online derivatization system, using 2-methyl-4-phenylaminomethylphenylboronic acid (2-methyl-4-PAMBA) as an immobilized derivatization reagent to extract BRs containing *cis*-diol and produce 2-methyl-4-PAMBA derivatives [143]. Using this strategy, six endogenous BRs were quantified in *Oryza sativa* L. cv., *Phaseolus vulgaris* L., *Vigna unguiculata*, and *Arabidopsis thaliana* flower. Carboxylic acid-metabolites (CCMs) are a large class metabolites that take part in the metabolite reprogramming in response to pathogen attacks [144]. However, their detection is rather challenging, attributed to the marked polarity differences, structural diversity, high structural similarity, and poor ionization efficiency in mass spectrometry [145]. Derivatization using 5-(diisopropylamino)-amylamine (DIAAA) also improved the detection of CCMs in Pu-er tea [146]. 

Acquisition in different ion modes negative, positive electrospray ionization (ESI), or atmospheric chemical ionization (APCI) can also overcome the limited ionization of certain metabolite classes and provide comprehensive metabolome coverage of the envisaged samples [147]. ESI is a commonly used ionization method in plant metabolomics profiling due to its ‘‘soft ionization’’ capability and the permission of direct analysis of biomolecules from the liquid phase, which is convenient for LC coupling [128]. APCI is in contrast a soft ionization method inducing little or no in-source fragmentation, which is suitable for LC-MS-based plant metabolomics. Besides, APCI is relatively tolerant to high buffer concentrations. ESI and APCI showed complementarity to each other, while ESI tend to ionize polar metabolites with a large molecular more efficient and APCI more suitable for non-polar and weakly polar metabolites ionization [148]. The comparison of ESI and APCI ionization in grape berry metabolites investigation showed that ESI was suitable for a wide range of metabolites including the weakly polar molecules such as flavanols, flavones, and acylated and glycosylated anthocyanins [149]. On the other hand, APCI showed better determination to polar metabolites, including sugars and organic acids. These results showed that more comprehensive profiling of plant metabolites could be achieved using ESI and APCI ionization modes in parallel.

The selection of mass detectors is according to the specific requirement of each study, in which high-resolution MS systems are required in untargeted metabolomics, while low-resolution MS systems are the workhorse for targeted metabolomics. For untargeted profile of phytoalexins and phytoanticipins, secondary orthogonal data are necessary for precise metabolite annotations [129]. In this case, tandem-in-time instruments, such as the Orbitrap and the Fourier-transform ion cyclotron resonance mass spectrometers (FT–ICR–MS), and tandem-in-space instruments including quadrupole time-of-flight instruments (qTOF), are usually used for untargeted plant–pathogen interaction metabolomics. The high resolving power, accurate detection of mass, and capability of MS/MS spectral generation enable the identification of a large number of metabolites associated with pathogen invasion (Table 1). In addition, triple quadrupoles (QqQ) and quadrupole linear ion trap (QTrap) are commonly used in targeted analysis of metabolites involved in plant metabolic reprogramming after pathogen exposure. Although these low-resolution detectors only provide nominal mass measurements, they enable the absolute quantification of metabolites. The acquisition types of neutral loss scan and precursor ion scan ensure the identification and quantification of metabolites [150], especially if targeting a certain class with a known fragmentation pattern or loss. With the optimization of multiple reaction monitoring (MRM) parameters using relative standards, highly selective and sensitive quantification of metabolites can be achieved. Targeted metabolomics analysis is widely used in plant metabolite responses to different stimuli induced by plant–pathogen interaction (Table 1). For example, the quantification of non-O-methyl and O-methylflavonoids in maize leaf elicited by *Bipolaris maydis* was analyzed by the Qtrap system, and a significant accumulation of non-O-methyl and O-methylflavonoids in infected middle leaf segments over the noninfected upper and lower leaf segments was observed, suggestive that these flavonoids are phytoalexins in maize [151]. However, the standards of some phytoalexins and phytoanticipins for MRM method establishment are difficult to obtain. Moreover, the internal standard used for peak intensities correction in absolute quantification, which is usually the heavy isotopes of the analysts, is expensive and not available for all metabolites. 

It should be noted that the plant sample must be adequately dissolved in the mobile phase and centrifuged at a very high speed (>15,000 rpm) before injection, in case of clogging of the chromatographic column. In addition, the low abundance of metabolites in the complex plant matrix is still the most challenging problem in LC-MS, which require optimized chromatographic separation to avoid the ion suppression effect induced by co-eluting interfering compounds. 

### 3.4. MS Imaging to Decode Spatial Changes in Plants Response to Stressors

Defensive compounds in response to stressors are of high specialization and are known to distribute in single- or few-cell structures [171,172]. However, in the chemical analysis of bulk tissue, the details of such distributions are lost during the extraction process, or require the cumbersome and multi-processed isolation of a given tissue for location [173]. MS imaging allows one to visualize the spatial distribution of thousands of primary and secondary metabolites on the sample surface (Figure 5). It can provide more comprehensive profiling including the spatial changes of plant defense and is increasingly reported nowadays in plant metabolomics studies [174]. 

There are several MS imaging techniques divided according to the way ions are generated. Secondary Ion Mass Spectrometry (SIMS) ionizes samples by ion bombardment while Matrix Assisted Laser Desorption Ionization (MALDI) uses laser illumination to generate ions with the use of a matrix, and Desorption Electrospray Ionization (DESI) uses an electrospray of charged solvent droplets to produce secondary ions [175]. 

SIMS was the first presented MS imaging technique. It is a hard ionization and is suitable for studying low molecular weight metabolites. Besides, the inherent fragmentation of large polymers into smaller units caused by hard ionization makes SIMS-based imaging able to determine stem and wood tissue for the distributions of saccharides and lignin units [176]. However, SIMS techniques can only detect the outer-most analytes of the sample and require the samples to be stable under a vacuum, which limits the application in plants.

MALDI is one of the most utilized methodologies for plant metabolites imaging owing for its flexibility, ease of use, speed, and well-documented sample preparation techniques. It is a soft ionization technique with little ion fragmentation [177]. This imaging technique has a non-targeting advantage for visual localization and to provide information on the production of phytoalexins and production action mechanism. For example, Abe et al. used the MALDI system to visually investigate the dynamic production of glyceollin phytoalexins in germinating soybeans inoculated with *Aspergillus oryzae* [178]. Results revealed that glyceollins were produced only inside th seed coat and germinated root of the soybeans, whereas their precursor, isoflavone, was distributed throughout the soybean. Therefore, it could be indicated that glyceollin phytoalexins are only produced in regions that are in contact with the fungus body. In addition, Seneviratne et al. used MALDI-MS for identifying metabolites involved in non-host disease resistance response [179]. Imaging data showed that pisatin and pinoresinol monoglucoside are accumulated and located in the endocarp tissue involved in the non-host resistance response upon exposure to *Fusarium solani* f. sp. *phaseoli* spores. This indicated that these metabolites played a role as phytoalexins during pathogen attack which also aided in understanding the function of DRR206, a protein that is involved in (+)-pinoresinol production. The location of phytoalexins makes sense for further understanding their function, biosynthesis, and possible transport within the plant. 

However, self-ionization of the organic matrix used in MALDI-MS occasionally interferes with ionizations of small molecules (<500 m/z), which limits its application in many plant phytoalexins measurement, including most plant hormones. To overcome such an obstacle, other techniques are developed, such as nanoparticle-assisted laser desorption/ionization (nano-PALDI). Nano-PALDI uses nanoparticles as an ionization matrix to avoid self-ionizing interference, which allows for the analysis of small molecules (m/z < 500) [180]. Seven common plant hormones and two associated compounds were used to compare the MALDI-MS and Nano-PALDI-MS detection efficiency [181]. Three of these compounds (auxin, brassinosteroid, D6- abscisic acid) failed to be ionized using MALDI-MS, whereas nano-PALDI-MS ionized all 9 chemicals in roots of rice (*Oryza sativa*). The ability of multiple major plant hormones visualization in plant tissues indicates the potential of nano-PALDI-MS in investigating the roles of hormonal signaling in stress responses. Besides, other MS imaging techniques are also utilized in plant–pathogen interaction. DESI is a technique that provides ionization under ambient conditions, which simplify sample pretreatment and obtain the high quality of needed information. Tata et al. used imprint imaging DESI-MS to monitor the dynamic of glycoalkaloids accumulation as phytoalexins in sprouted potatoes infected by *Pythium ultimum* [182]. In total, 10 potato glycoalkaloids were determined, two of which (α-solanine, α-chaconine) showed decrease after 8 d infection, while others showed an increase suggestive of different roles inside tubers. With disease progression, all glycoalkaloid metabolites showed a decrease trend. These results proved that DESI-MS could efficiently decode for metabolic changes after a pathogen attack in plants, which could be a powerful technique for plant–pathogen interaction studies in the future yet to be more capitalized upon. 

## 4. Metabolomics Data Analysis and Visualization

**Figure 7:**
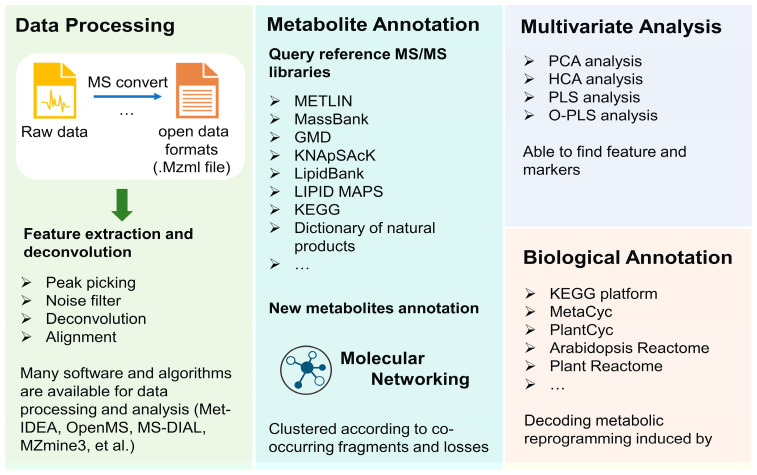
General data analysis procedure in metabolomics reprogramming investigation as typical in studying pathogen–plant interactions.

NMR- and MS-based strategies typically generate a large amount of data which makes data analysis a great challenge in untargeted analysis for the visualization and further markers identification. Although there is no standard protocol for data analysis, generally data analysis can be divided into several steps: data processing, metabolite annotation, quantification, and statistical analysis (Figure 7). 

The purpose of data processing is to extract spectra and make data clearer, which further aids in metabolites annotation and mapping to specific pathways. It usually includes several steps. However, for MS data, the conversion of original proprietary formats into open data formats (mzXML, mzData, mzML, netCDF, et al. [183,184]) is necessary prior to their analysis. These formats are readable in most standard statistical environments and are convenient for further analysis. Feature detection is the first step; the low-intensity metabolites can be separated from noise-by-noise filter algorithms, and the co-eluting metabolites can be unraveled during the deconvolution step. After the operating peak alignment algorithm, the metabolite features between different chromatographic runs can be compared and normalization methods lay the fundamental for quantitative metabolomics analysis. Up to now, many platforms can provide reliable data preprocessing of plant metabolomics, including Met-IDEA [185], OpenMS [186], MS-DIAL [187], and MZmine3 [188,189]. In addition, Metaboanalyst 5.0 [190,191], web tool-integrated and untargeted LC–MS spectra processing, functional analysis, and functional meta-analysis for multi-omics analysis, can not only process raw data but also carry out statistical analysis, which are effective tools in metabolic analysis involved in plant–pathogen interactions [192,193]. 

Identification of metabolites is the vital part for data analysis [194]. The annotation is always based on MS1 and MS2 data with the combination of databases including pathway-centric databases and compound-centric databases [128]. METLIN and MassBank are comprehensive databases that are available for plant metabolomics analysis [195]. Other databases, such as GMD, KNApSAcK, LipidBank, LIPID MAPS, KEGG, and PlantCyc, are also utilized for metabolites annotation though still generalized and not specific per plant genotypes. Major challenges in secondary metabolites annotation in plant taxa lie in the huge diversity in strictures and metabolite classes as compared with human or animal databases and which is still the major bottleneck in all plant metabolomics projects. Plant databases which are more specific to serve for plant metabolite analysis are necessary for plant metabolomics studies. For example, the Dictionary of Natural Products, AntiBase, and MarinLit are commonly used for the identification of known compounds in plants. Besides, other new plant-specific databases also showed their powerful potential in plant metabolomic metabolomics analysis. ReSpect (RIKEN tandem mass spectral database) represents another potential plant specific MS/MS-based database, established based on published data and standard compounds [196]. A plant natural product tandem mass spectral library was constructed by Lei et al., in which most focused on plant phenolics such as flavonoids, isoflavonoids, and phenylpropanoids [197]. Global Natural Products Social Molecular Networking (GNPS) is a natural product and metabolomics analysis database, which can analyze a dataset and compare it to all publicly available data [198]. The networking approach can also aid in identifying unknown structures based on their clustering with known ones [199]. Moreover, some more specific databases focusing on model plants are also increasingly developed. For instance, mass spectrometry databases for *Arabidopsis* developmental (AtMetExpress) [200], ecotype [201], and mutants (PlantMetabolomics [202], MeKO [203]) were developed and available for metabolites annotation and metabolomics dynamic analysis. Application of plant-specific databases in plant microbe interaction is less explored in the literature and has the potential to aid in identification of markers for infected plant organs, etc., based on visual inspection of pie chart areas for the different samples, i.e., control versus infected ones. In addition, with the development of plant–pathogen interaction investigation, some databases constructed by metabolites involving in plants’ response to stressors should be established. Researchers can update their data in the public data repository, such as GNPS/MassIVE [204], Metabolomics Workbench [205], and MetaboLights [206]. A broad range of experimental methods and conditions in public open data can support effective metabolomic profiling. Therefore, data depositions from metabolomics researchers across the world are essential for metabolic studies and should be mandated at some point upon publishing, especially for metabolomics reprogramming in plants, the field of which still requires a richer set of data types. The broadening of public datasets may also facilitate the sharing of worldwide data and foster collaborations for metabolomics reprogramming studies in the future, especially to compare field studies from different regions worldwide. It should be noted that the identification procedure of metabolites should reference the metabolomics standards initiative to prevent the risk of poor-quality control and deceptive data interpretation mostly in the metabolites identification part [207]. The standards facilitate the data obtained by different analysis methods and from different biological systems to be available to others for evaluation, extension, or sharing in a public repository [208]. In addition, there are several types of in silico fragmentation software for compound identification according to mass databases, including MetFrag and SIRIUS 5. MetFrag implements compound identification based on compound database searching and fragmentation prediction [209], while SIRIUS 5 provides coherent assessment of molecular structures according to the combination of isotope pattern analysis and fragmentation trees [210]. These tools have shown their efficiency in many metabolic analyses of plants invaded by pathogen and significantly benefited the untargeted profiling approach [211,212,213].

Moreover, there are many new metabolites involved in metabolites reprogramming that have yet to be discovered mostly due to the lack of standard MS/MS spectra. The annotation of these metabolites is also urgent. Instead of comparing them with the reference spectra, strategies that annotate metabolites according to co-occurring fragments and losses were established to address this problem [214]. GNPS allows one to build a molecular network based on the similarity of mass spectra (generated by structurally related metabolites), and to annotate the unknowns based on the molecular [198]. According to feature-based molecular networking (an integrated tool in GNPS) and the characteristic ion of ascorbic acid, Zhang et al. identified 17 ascorbic acid derivatives in the fruit of *Rosa roxburghii* Tratt [136]. These derivatives showed the interaction between ascorbyl acid and organic acids, flavonoids, and glucuronic acid. Similarly, MetDNA also utilizes a recursive algorithm for annotating unknown metabolites. Metabolites are identified based on their similarity of MS/MS spectra and possible metabolic relations. It has been proven that this strategy could annotate nearly 2000 metabolites based on three spectral databases of metabolites [215]. Although metabolites annotation is convenient with the development of databases and computational tools, identification of isomers and the influence brought by in-source degradation products are still challenging [216]. Expertise in data extraction steps prior to modelling can also overcome problems such as in-source degradation products aided by tools such as Mzmine [217] that can avoid their inclusion in the dataset to be modelled.

Furthermore, multivariate analyses are used to visualize datasets in a general untargeted manner and to aid in reducing the dimensionality of the data. Multivariate analysis methods account for potential trends in complex data sets, which aid to interpret pathogens-induced metabolomic perturbations and reprogramming seen in the system under study. Unsupervised methods, such as Principal component analysis (PCA) and hierarchical clustering analysis (HCA), supervised discriminant techniques, such as partial least squares discriminant analysis (PLS-DA), and orthogonal to partial least squares discriminant analysis (OPLS-DA), are usually preferred for multivariate analysis, which can determine class-based differences between experimental groups. However, the overfitting of the model is a main question for poor quality and predictive performance of the models developed [218]. In this case, researchers are usually utilizing a combination of statistical approaches to select representative changes in metabolome and find biomarkers related to pathogen–plant interaction. For example, Finnegan et al. used OPLS-DA combined with VIP (variable importance in projection) plot analysis to identify biomarkers that contributed to metabolomic reprogramming induced by LPS [135]. In this study, a total of 106 biomarkers (64 of them in negative mode acquisition, 42 of them in positive mode acquisition) were identified.

The data analysis of NMR data is similar to that of MS data, including data processing, spectra annotation, quantification, and statistical analysis, though with still some differences in processing. In data processing, alignment, baseline correction, bucketing (binning), normalization, and scaling are involved. Alignment using internal standard or computational approaches removes the interference leading by pH, temperature, salt concentration, and inhomogeneous magnetic fields. Baseline correction shields the noise and selects signals only from the metabolites. Bucketing allows for moderate shift averaging at the expense of resolution and provides a matrix for further processing [219]. Normalization aids for removing systematic errors, whereas scaling helps for low abundance metabolite analysis, though scaling is less problematic in the case of NMR as it has an inherent equal metabolite response compared to MS detection. Metabolites annotation is also an important part in NMR data analysis. The lack of comprehensive NMR spectroscopy databases that focus on plant metabolites makes the interpretation of NMR data require the prior study of possible structure of metabolites and tedious work [101]. Many databases such as Chenomx NMR Suite, Bayesil, and COLMARm are used for metabolites references to reduce the complicated annotation procedure and avoid the dereplication of identification of known metabolites. In addition, some plant-specific NMR databases are also developed, including MeRy-B [220] and MetIDB [221]. Especially, MetIDB is a reference database specific for flavonoids. It contains 6000 ^1^H NMR spectra of flavonoids, which may facilitate flavonoids profiling involved in metabolomics reprogramming after pathogen attack. Moreover, one of the most important advantages of NMR spectra is the highly accurate and reproducible quantification ability. For metabolites quantification, internal standards with unique and simple structure, such as sodium trimethylsilylpropanesulfonate (DSS), sodium trimethylsilylpropionate (TSP), etc., are added at a known concentration for comparing the NMR peak heights with target metabolites. For example, the TSP-d4 standard was used to assess metabolites dynamic of xylem after explosion of *Ralstonia solanacearum* [110]. The quantification revealed that glutamine (and asparagine) are primary resources for *Ralstonia solanacearum* during its colonization phase. 

The overall process above tends to interpret ultimate biological results or end products, with less emphasis on decoding metabolic reprogramming in plant innate immunity, and the elucidation is quite necessary and aids in identifying changes in intermediates more difficult to be detected [222]. To pinpoint altered metabolic pathways, many platforms are available; KEGG is the most popular database for metabolome mapping on biosynthetic pathways. For instance, Zhu et el. investigated metabolic changes in hypocotyls of two soybean lines after *Phytophthora sojae* infection [119]. With the help of the KEGG database, altered metabolites belonged mostly to sugar, organic acids, amino acid derivatives, and other secondary metabolites participated in the metabolic-level defense response of soybean to *P. sojae*. Except for the KEGG platform, some plant-specific database platforms are emerging to support plant metabolomics systematic analysis, including PlantCyc, TritiCyc, Arabidopsis Reactome, and Plant Reactome [223]. 

## 5. Conclusions and Future Perspectives

Better understanding of plant–pathogen interaction is one of the pivotal tools needed to deal with agricultural sustainability as well as food security. In this review, we presented an overview of the major plant responses to biotic stresses at the metabolite level. The defensive role of these specialized metabolites, i.e., phytoalexins in response to pathogen attack has been described. In addition, the current analytical technologies used for decoding metabolic reprogramming in pathogen–plant interactions, viz., NMR, GC-MS, LC-MS, and imaging mass spectrometry were reviewed with comparison among techniques, clarifying for each technique its applications, detection towards which metabolite classes, advantages, and any limitations. Both ^1^H and 2D NMR spectroscopy have been widely employed to decode metabolome that might be implicated in plant defense against pathogen attacks considering NMR universal detection, though less sensitive compared to MS-based metabolomics. Cryogenic NMR probes allow a drastic enhancement of the sensitivity in NMR experiments leading to either an essential reduction of experiment times or a reduction of the required sample amount. Additionally, other NMR cutting edge techniques such as high-resolution solid-state magic angle spinning (HR-MAS) NMR could also be beneficial for analysis of semi-solid samples such as fresh plant leaves or intact tissues under biotic stresses. On-line hyphenation of separation techniques and NMR spectroscopy such as HPLC-NMR and more recently, LC-SPE-MS-NMR represent another potential technology that can be applied for the de novo identification of metabolites in response to pathogen attacks, especially considering the stronger structural elucidation power of NMR compared with MS technology. Regarding sampling and analysis of VOCs, use of ionic liquids as sample preparation technique shows excellent capturing performance in the face of VOCs and can be employed in sampling of these metabolites following pathogen attacks less reported in the literature. Another cutting-edge technology employed for the online capturing and analysis of VOCs include proton transfer reaction mass spectrometry (PTR-MS) and laser-induced breakdown spectroscopy (LIBS). Unlike GC-MS, such technologies can aid in monitoring and detection of VOCs following pathogen attacks with real-time monitoring capabilities and moreover, at a high sensitivity level. 

LC-MS is a powerful technique for metabolomics analysis, especially for secondary metabolites (alkaloids, flavonoids, terpenoids, etc.) determination, which is quite difficult to be analyzed in NMR and GC-MS investigation. The efficient separation facilitates the identification of low-abundance secondary metabolites. Different column technologies pose LC-MS to analyze metabolites with different polarities. For metabolites with poor retention in the LC system and/or poor ionization efficiency, strategies such as ion-pairing and derivatization can improve their detection. Different acquisition modes and ion sources can also achieve better ionization of specific metabolites and provide a global metabolomics profile of the envisaged plant sample. In addition, the spatial distribution of metabolites involved in stress response observed by MS imagining is also necessary for metabolomics reprogramming investigation. Asides from the commonly used MALDI technique, some newly developed techniques, such as Nano-PALDI and DESI, were employed to avoid the interference of self-ionization and simplify sample preparation. Data analysis and visualization is challenging due to the large amount of datasets acquired by NMR and MS based analysis. Metabolites annotation is a vital step among data analysis, with many databases being continuously developed to meet such a goal and assist metabolites identification, as exemplified by some plant-specific databases, including the Dictionary of Natural Products, ReSpect, and GNPS. For NMR data annotation, some plant-specific databases are also emerging, such as MeRy-B and MetIDB. For those unknown metabolite annotations, a feature networking strategy and recursive algorithm have been developed to aid in their identification, being the major bottleneck in plant metabolomics studies and more specifically, in plant microbe interactions. Furthermore, multivariate analyses help to identify representative biomarkers in metabolomics dynamics induced by pathogen attacks, which provide evidence to account for the mechanism of plants’ stress response towards immunity or in overcoming disease progression. Pathogens can perturb plants’ physiological processes though the secretion of effector molecules that inhibit plant defense mechanisms or the availability of nutrients. Effectors are mostly proteins, but other non-protein metabolites like coronatine, which is secreted by *Pseudomonas syringae*, provoke metabolic perturbations in infected plants, facilitating the pathogen entrance to host tissues. Metabolomics can contribute to understanding pathogen-associated molecular changes in the infected plants caused by effector molecules. Intriguingly, the recent analytical developments such as mass spectrometry imaging (MSI), matrix-assisted laser desorption ionization (MALDI), and live single-cell mass spectrometry (LSCMS) can allow the discrimination of plant-specific metabolites from those produced by pathogens, thus enabling the pathogenesis control which is crucial in developing sustainable crops in the future.

## Figures and Tables

**Table 1:** The commonly used mass detectors applied in plant–pathogen studies.

Mass Spectrometer Technique	Approach	Altered Metabolites	References
qTOF	Untargeted	Oxylipins, phenolic lipids, diacylglycerol, phosphatidic acid	[152]
	Untargeted	Phytohormones salicylic acid, jasmonic acid, indole derivatives, phenylpropanoids	[135]
	Untargeted	Phenolic amino acids, phenylpropanoids, hydroxycinnamic acid amides, fatty acids, lysophospholipids, glycoglycerolipids, and phospholipids	[153]
	Untargeted	Oxylipin, amino acids	[154]
	Untargeted	L-Glutamate, DIBOA-glucoside, fatty acids, phospholipids, flavonoids, carotenoids, and alkaloids	[155]
Ion-trap	Untargeted	Polyphenolics	[156]
	Untargeted	Terpenoids, phenylpropanoids, flavonoids	[157]
	Untargeted	Arabidopsides	[158]
	Untargeted	Polyphenolics	[159]
Orbitrap	Untargeted	Aldehydes, alkaloids, carboxylic acids, flavonoids, phenolics	[160]
	Untargeted	Carboxylic acids, flavonoids	[161]
	Untargeted	Carboxylic acids, flavonoids, amino acids, sugars	[162]
	Untargeted	Amino acids, fatty acids, phenylpropanoids	[163]
	Untargeted	Amino acids, carbohydrates, phenylpropanoids, terpenoids	[164]
FT–ICR–MS	Untargeted	Phenolics, alkaloids, carboxylic acids	[165]
	Untargeted	Flavonoids, carboxylic acids	[166]
QqQ	Targeted	Oxylipins	[167]
	Targeted	Polyamines	[140]
	Targeted	Isoquinoline alkaloids	[139]
	Targeted	phenylpropanoids, benzoic acids, glycoalkaloids, flavonoids, amino acids, organic acids, oxygenated fatty acids	[168]
Qtrap	Targeted	Terpenoids	[169]
	Targeted	Flavonoids	[170]
	Targeted	Flavonoids	[151]
